# Online characterisation of oxidative potential and reactive oxygen species of fresh and aged aircraft turbine and ship engine particulate emissions

**DOI:** 10.1039/d5ea00174a

**Published:** 2026-09-22

**Authors:** Alexandre Barth, Battist Utinger, Quanfu He, Andreas Paul, Anni Hartikainen, Mika Ihalainen, Uwe Etzien, Sandra Piel, Marius Rohkamp, Ajit Mudan, Benedikt Gündling, Kokkola Tuuka, Thorsten Streibel, Martin Sklorz, Hendryk Czech, Johan Øvrevik, Thomas Adam, Andreas Hupfer, Thorsten Hohaus, Olli Sippula, Yinon Rudich, Bert Buchholz, Ralf Zimmermann, Markus Kalberer

**Affiliations:** a Department of Environmental Science, University of Basel Basel Switzerland markus.kalberer@unibas.ch; b Institute for Climate and Energy Systems, ICE-3 Troposphere, Forschungszentrum Juelich GmbH Juelich Germany; c Department of Environmental and Biological Science, University of Eastern Finland Kuopio Finland; d Department of Piston Machinery and Internal Combustion Engines, University of Rostock Rostock Germany; e Institute for Chemistry & Environmental Engineering, University of the Bundeswehr Munich Neubiberg Germany; f Department of Technical and Analytical Chemistry, University of Rostock Rostock Germany; g Cooperation Group “Comprehensive Molecular Analytics”, Helmholtz Centre Munich Munich Germany; h Norwegian Institute of Public Health Oslo Norway; i Department of Environmental and Biological Science, University of Eastern Finland Kuopio Finland; j Department of Chemistry, University of Eastern Finland Joensuu Finland; k Department of Earth and Planetary Science, Weizmann Institute of Science Rehovot Israel; l Institute for Aeronautical Engineering, University of the Bundeswehr Munich Ottobrunn Germany

## Abstract

Anthropogenic particulate matter (PM) emissions from the transport sector are a major contributor to global air pollution and associated adverse health effects. While the health impacts of PM are well-documented, the specific chemical and physical properties driving its toxicity, such as oxidative potential (OP) remain poorly understood and quantified. This study investigates the OP activity of PM emissions from a small jet engine combustion chamber (using JP-8 fuel) and a marine diesel engine (using low sulfur Heavy Fuel Oil, LS-HFO, and Marine Gas Oil, MGO) under fresh and photochemically aged conditions. Using a novel online instrument the Online Oxidative Potential Ascorbic Acid Instrument (OOPAAI)—we quantified in real-time OP activity of PM. Fresh emissions were sampled directly, while the aged emissions were processed in a photochemical reactor to simulate atmospheric ageing for 1 to 12 equivalent days. Our results illustrate that mass-normalized OP activity showed no significant change with particle ageing for ship emissions but increased substantially for aircraft emissions. The average chemical composition of PM, characterized by average O/C and H/C ratios of the organic mass fraction, indicated increasing oxidation with ageing, correlating well with higher OP activity of aircraft emissions. These findings highlight the importance of considering both fresh and aged emissions in assessing the health impacts of PM emission from fossil fuel sources, as atmospheric processing can drastically alter their oxidative properties.

## Abstract 2

Freshly emitted and aged anthropogenic particulate matter emissions from the transport sector (shipping and aircraft) contribute significantly to particle oxidative potential, a particle toxicity indicator.

## Graphical abstract

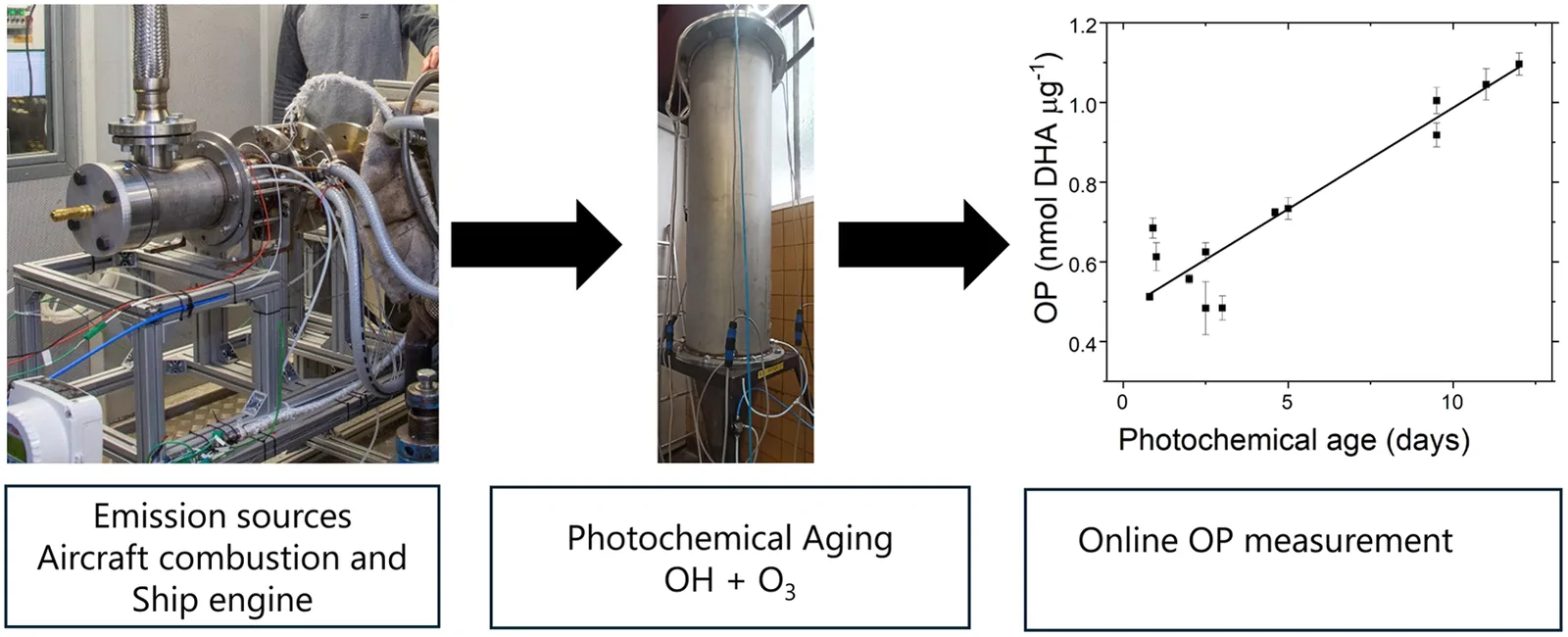

**Environmental significance**

Particulate matter (PM) emissions from the aviation and maritime transport sectors contribute substantially to global air pollution and associated health risks. However, current regulations primarily target total PM mass, without considering possible toxicity-relevant PM properties such as oxidative potential (OP). This study demonstrates that atmospheric oxidative ageing enhances the oxidative characteristics of aircraft emissions, and suggest that existing emission standards may not fully account for toxicity-relevant particle properties.

## Introduction

After decades of epidemiological research, it is clear that air pollution is linked to a wide range of adverse health effects such as cardiovascular, respiratory diseases or cancer.^1,2^ An estimated 6.7 million pre-mature deaths per year can be attributed to elevated levels of air pollution.^3^ Particulate matter (PM) is the most toxic component in polluted air even in low pollution areas like in many European countries.^4^ PM has a wide range of sources both biogenic and anthropogenic^5,6^ but the anthropogenic contribution to PM is of more concern for public health due to its higher toxicity.^7,8^ PM can be emitted directly into the atmosphere from sources like combustion of fossil fuels, industrial activities and non-exhaust traffic emissions as primary PM.^9–11^

Emissions from the shipping industry also noticeably contribute to ambient PM, affecting especially local pollution levels, *e.g.* close to harbors,^12,13^ contributing up to 15% of total PM_2.5_ in European coastal regions.^14^ The situation is similar in other major coastal regions in the world with up to 25% of PM_2.5_ originating from the shipping industry in East Asia.^15^ Indirect effects, *e.g.*, caused by large amount of NO_*x*_ emissions can lead to a 2 to 3 fold increase in PM concentration in marine areas.^16^ Particulate ship emissions have also been specifically linked to increase the risk of cardiovascular disease and are attributed to up to 0.5% of global mortality.^17,18^

Emissions from other sources in the traffic sector like aircraft emissions may also play an important role in affected areas.^19^ Aircraft emissions can cause locally increased exposure to PM around an airport due to high PM concentrations and large exhaust volumes.^20^ Daily mean PM concentrations can be significantly higher in the vicinity of airports than at background sites.^21^ In airports close to major urban areas, aircraft emissions could be relevant in the assessment of potential public health consequences.^22^ It can be assumed that aircraft emissions can have adverse health effects on a similar level as diesel exhaust which is a significant health risk for airport personnel, passengers, and local residents.^23^ The monetized value of the mortality and morbidity effects caused by landing and take-off (LTO) emissions was estimated at 1.4 billion USD in the US alone.^24^

Besides primary emissions, oxidation processes in the atmosphere involving gaseous organic and inorganic precursors can produce secondary PM. In urban environments, anthropogenic secondary organic aerosols (SOA) can contribute between 17 and 39% of PM_1_.^25^ Both ship and aircraft gaseous emissions have high potentials to form secondary aerosol. Especially under idling conditions of aircraft engines the formation of SOA is orders of magnitude higher than primary emissions^26,27^ and SOA from shipping emissions are also a major source of inorganic secondary particles containing sulphate and nitrate.^28^

The chemical and physical properties of PM leading to the negative health effects as well as the toxicity mechanisms still remain largely unknown.^29,30^ One of the suggested toxicity mechanisms, an increase of oxidative stress in the lung and systemically, is postulated to be a common factor for the adverse health outcomes of PM exposure,^31^ leading for example to cardiovascular diseases.^32,33^ A metric that might explain particle induced oxidative stress is the oxidative potential (OP) of PM that refers to the production of reactive oxygen species (ROS) in the lungs upon exposure. It is caused by PM constituents like quinones, polyaromatic hydrocarbons (PAHs), redox active transition metals (*e.g.* iron, copper), radicals, and inorganic and organic peroxides. ROS can also be present in PM before inhalation.^34^ Traditionally, OP measurements are based on filter collection of aerosol particles and offline analysis methods. However, techniques measuring OP online and continuously offer many advantages. Fast OP changes due to the dynamic nature of some sources can be fully observed and the workload to obtain long times series of OP or ROS is greatly reduced with online techniques. Recent studies have shown that there can be a large difference between online and offline OP reactivity where sample degradation was observed and up to 99% of the online signal is lost within days when using offline techniques.^35^

In this study we investigated primary and secondary PM originating from a combustion chamber of an aircraft turbine as well as from a marine diesel engine as part of the ULtrafine particles from Transportation – Health Assessment of Sources (ULTHRAS) project.^36^ While the turbine was running with one fuel (JP-8) the marine engine was operated with two fuel grades, low-sulphur heavy fuel oil (LS-HFO) and marine gas oil (MGO). In addition to measuring fresh emissions directly at the source, they were also passed through an oxidation flow reactor to investigate different photochemical ages and simulate atmospheric oxidation and photochemical ageing processes and determine their influence on OP and ROS of PM.^37^ With our measurements we quantify OP and ROS activity of PM by deploying two recently developed online instruments^38,39^ that capture fast changes of OP and ROS characteristics.

## Methods

Two anthropogenic PM sources from the transport sector were investigated. To represent emissions from an aircraft, the combustion chamber of a small jet engine without additional compression stage was operated.^40,41^ Despite being primarily used for the propulsion of small, unmanned vehicles, the composition of gaseous emissions is representative of real-world emissions of older-generation gas fan engines as described in Hartikainen *et al.*, 2025.^41^ The combustion chamber was set to a constant load of 7% simulating an average value for the standard landing and take-off cycle which includes ground operation as well as take-off and approach of an aircraft. The combustor rig was running on JP-8 fuel which is used in military aviation in NATO air forces. It is identical to Jet A-1 except for specific additives like lubrication improvers, corrosion inhibitors, and static dissipaters to enhance its performance.^42^ Jet A-1 is one of the most commonly used fuel in commercial aviation.^43,44^

Furthermore, a four-stroke single-cylinder marine research engine^45^ with a displacement of 3.18 L and a rated power of 80 kW at 1500 rpm using MGO (Marine Gas Oil) and LS-HFO (Heavy Fuel Oil) fuel was operated to study shipping emissions. The MGO used had a sulphur content of 0.015% while LS-HFO had 0.5% sulphur (more information on fuel properties is given in Table S1). The shipping industry uses a range of fuel grades that differ in PM and SO_2_ emissions.^46^ Typically, LS-HFO is used as the most economical option on the open sea. Inside sulphur emission control areas where ships operate near coasts like in the North Sea and Baltic Sea the fuel sulphur content limits are stricter only allowing for 0.1% sulphur.^47^ The ship engine used here featured an adjustable external charge air compression system and a common rail injection system and NO_*x*_ emissions were set to be compliant with Tier II in IMO regulation 13. These emissions standards are valid for ships built between 2011 and 2021.^47^ The same engine has been used in previous studies,^48–50^ where gaseous and particulate emissions have been characterized, for example aromatic hydrocarbon profiles in the gas phase and size and composition of aerosol particles. For direct emission analyses (defined here as “fresh emissions”), the ship engine was operated following a variable load cycle increasing from 25% to 50%, 75% and 100% (20, 40, 60, 80 kW power output) and then decreasing stepwise again to 25% with a total duration of the cycle of 4 hours, as shown in Fig. S1. 75% Load represents optimal fuel consumption and is the usual load during transport operation. Before each cycle, the engine was preheated briefly at 50% load to avoid any changes due to the engine warming up during the cycle.

Engine emissions were collected from the raw exhaust stream *via* a two-stage dilution system using a porous tube and ejection diluter to cool the emissions down and establish a stable dilution ratio for all measurements. The dilution ratio and stability were monitored by measuring CO_2_ concentrations before and after the dilution system. Different dilution ratios were necessary to satisfy particle concentrations needed of the different instruments operated during these experiments^41^ (Table S2).

**Fig. 1:**
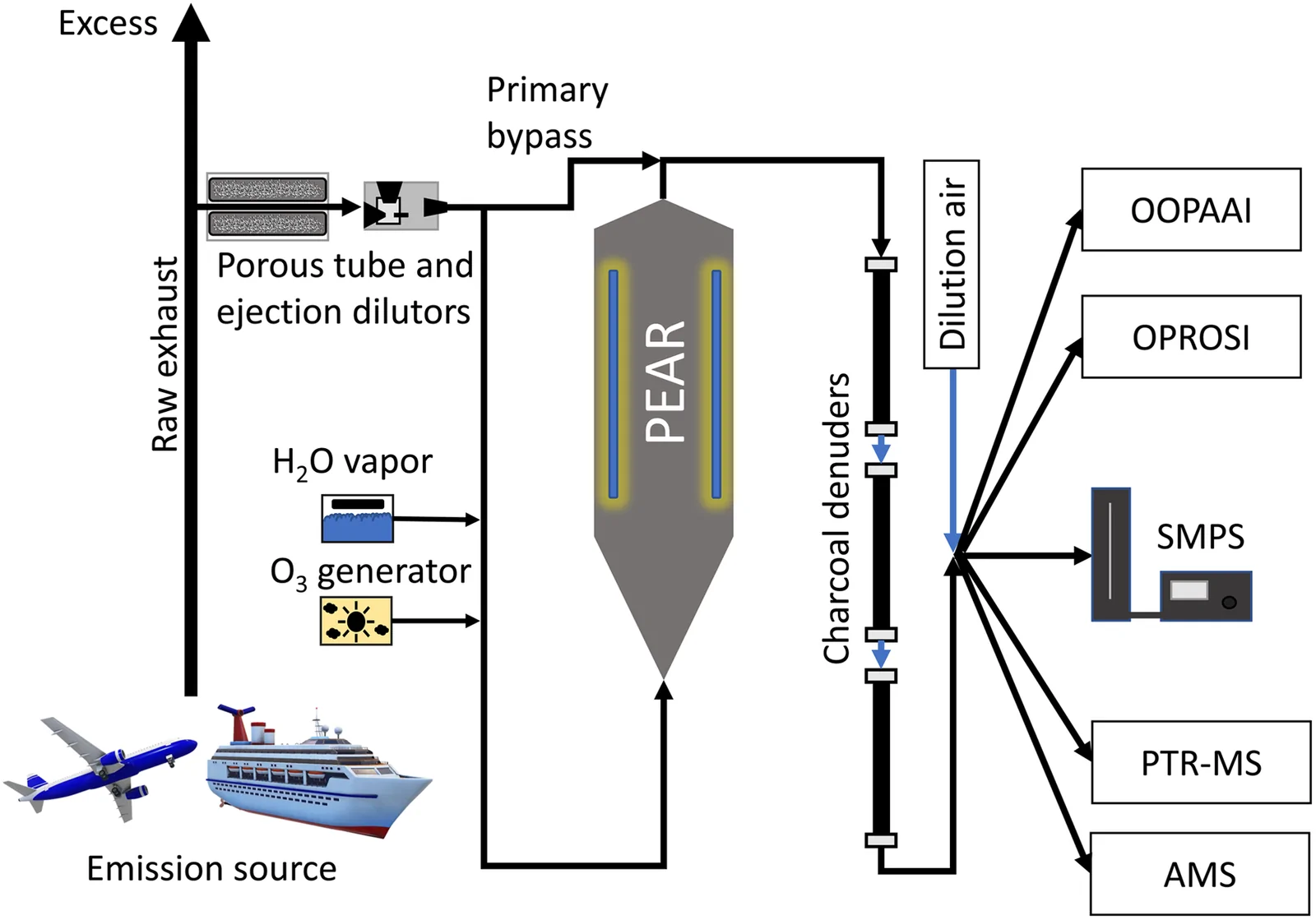
Simplified schematic of the experimental setup. After diluting the sample from the raw exhaust line, it was either analysed directly for OP and ROS (primary bypass) or passed through the PEAR which simulated atmospheric aging.

After dilution, the sample flow was either analysed directly as fresh emissions or passed through the Photochemical Emission Ageing flow tube Reactor (PEAR)^51^ (Fig. 1), simulating chemical and physical changes of the exhaust particles during atmospheric ageing with exposure to UV light, OH and O_3_. By mixing O_3_ (10 ppm) and water vapour (50% relative humidity) into the sample stream at the inlet of the PEAR, equipped with UV lamps (peak emission at 254 nm), a steady concentration of OH radicals is generated.^51^ The aerosol residence time in the PEAR was about 70 s, thus no significant physical or chemical changes are expected due to the slightly longer time between emissions and analysis when particles were passed through the PEAR compared to the primary bypass. To determine the OH concentration and the related photochemical age, a constant flow of *d*_9_-butanol was added^52^ and its decay caused by OH oxidation was monitored by a Proton Transfer Reaction Time of Flight Mass Spectrometer (PTR TOF-MS 8000, Ionicon), as described in more detail in Paul *et al.*, 2025.^53^ Integrated OH concentrations, equivalent of up to 12 equivalent days (eq. days) photochemical ageing, were simulated in the PEAR, assuming typical tropospheric OH concentrations of 1.5 × 10^6^ molecules cm^−3^. It should be noted that oxidation flow reactors such as the PEAR might lead to a different molecular distribution of oxidizing particle components compared to aging in the ambient atmosphere, due to much higher oxidant concentrations in the PEAR and the short residence time, which might affect especially slowly forming products such as hydroperoxides formed in autooxidation reactions.

The main focus of this study was to quantify OP of the exhaust particles using a home built automated, online instrument. The Online Oxidative Potential Ascorbic Acid Instrument (OOPAAI) quantifies the oxidation of ascorbic acid (AA) to estimate particle OP.^38^ The OOPAAI samples air at a flow rate of 15 L min^−1^ in a Particle-Into-Liquid-Sampler (PILS, Brechtel). A wash flow of 60 µl min^−1^ in the PILS containing 200 mM AA in an aqueous 20 mM HEPES ((4-(2-hydroxyethyl)-1-piperazineethanesulfonic acid) buffer continuously washes the particles off an impaction plate a few seconds after they enter the instrument where the PM-induced oxidation of AA starts. The liquid flow then passes through a heating bath at 37 °C for 20 min. Afterwards, 90 µl min^−1^ of 20 mM *o*-phenylenediamine (OPDA) is added reacting with dehydroascorbic acid (DHA), the main oxidation product of AA, to produce the fluorescent product 1,2-dihydroxyethyl)-fluoro[3,4-*b*]quinoxaline-1-one (DFQ), which is quantified *via* fluorescence spectroscopy (excitation: 365 nm; detection: 10 nm integration window between 425–435 nm^38^). DHA was used as calibrant and therefore, OP activities are given in units of “nmol DHA per µg PM mass”. A second instrument (Online Particle-bound Reactive Oxygen Species Instrument (OPROSI)) with a similar working principle, but with a different assay (HRP/DCFH (horseradish peroxidase/2′7′-dichlorofluoroscein)) was used to obtain supporting and complementary data.^39^

All solutions were prepared the evening before the measurements. To establish a background level, particle free blanks were measured by passing sample air through a HEPA filter (HEPA-CAP150, Whatman) before, during and after each measurement day. In front of the instrument two 1 m long home-built charcoal-filled (activated, untreated, granular, Sigma-Aldrich) denuders as well as 8 honey comb-shaped charcoal denuder elements (Ionicon) were connected in line at room temperature. The denuders removed any OP- and ROS-active gas phase components (*e.g.*, O_3_, NO_*x*_, oxygenated VOCs) that could interfere with the instruments, as confirmed with the frequent blank measurements (at least every 4 hours). Particle mass losses of *ca.* 45–55% in the series of denuders were determined by measuring the particle size distribution before and after the denuders (Fig. S2). The significant particle losses in the denuder might have introduced some measurement uncertainties if different particle sizes would be differently OP active. Such a potential uncertainly could not be assessed with the current experimental set up, as this would require size-segregated OP measurements. Additionally, to counter low inlet pressures a clean air dilution was added after the denuders (1–5 L min^−1^, Fig. 1).

For the aircraft emissions particle number size distributions were determined with a TSI Scanning Mobility Particle Sizer (SMPS 3938, TSI) and for the ship emissions with a Grimm SMPS (SMPS + C 4520, GRIMM). Particle densities used for the particle mass calculations were determined by Hartikainen *et al.*, 2025,^41^ and Paul *et al.*, 2025,^53^ for the aircraft and ship emissions, respectively. An average of 1.35 g cm^−3^ was used for the aircraft particles while 1.5 g cm^−3^ was used for the ship experiments. Overall particle composition indicators such as O/C and H/C ratios were determined using a HR-ToF-AMS (High-Resolution Time of Flight Aerosol Mass Spectrometer, Aerodyne), as detailed in Paul *et al.*, 2025.^53^

## Results and discussion

OP activity of two distinct anthropogenic PM sources were investigated. While the ship engine followed a load cycle to cover different operation modes, the aircraft combustion chamber was operated at a steady load to represent average overall emissions of an LTO cycle. Besides the sampling of fresh emissions, particles were also passed through the PEAR to simulate atmospheric ageing to investigate age-dependent changes of particle oxidising properties.

### Ship engine

**Fig. 2:**
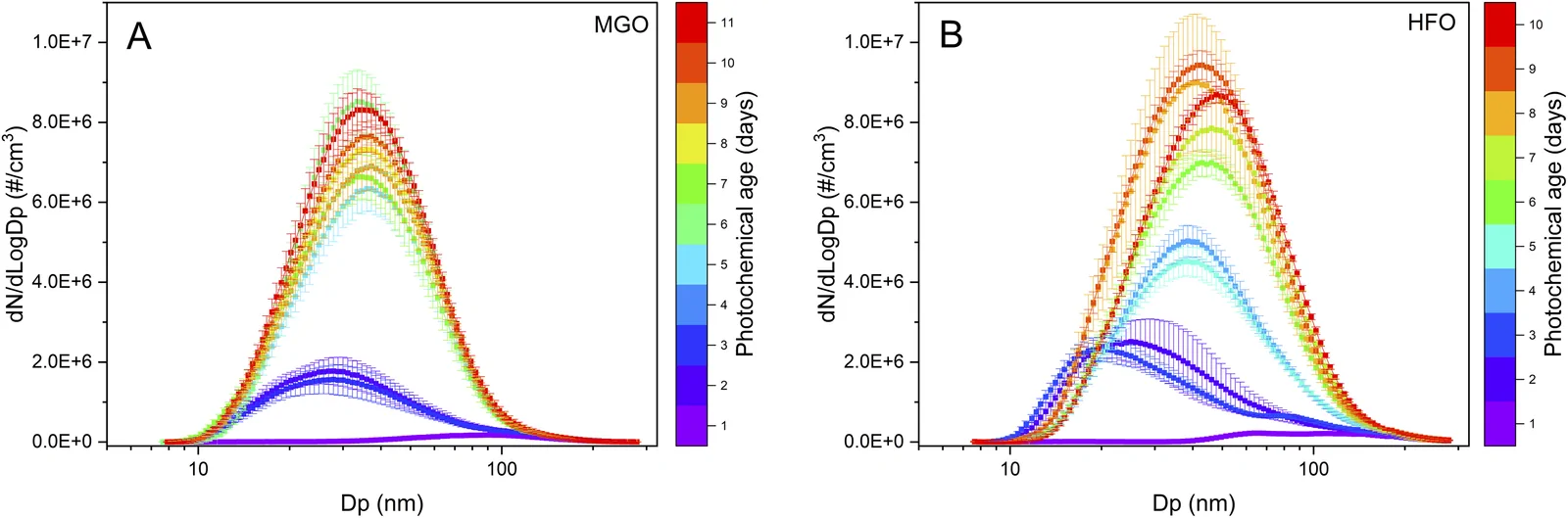
Particle size distributions during ageing experiments of ship engine emissions operated with MGO (A) and LS-HFO (B) fuel for an equivalent of 1 to 11 eq. days atmospheric ageing.

For fresh engine emissions, no significant difference was observed in particle size and number between the two fuels MGO and LS-HFO although a large variability in the size distribution was measured due to variations in engine performance (Fig. S3). Increasing photochemical ageing, however, lead to a strong increase in the particle number size distributions (Fig. 2A and B) compared to short ageing times or fresh emissions, which is due to new particle formation from gaseous precursors being oxidised in the PEAR. At low ageing conditions, equivalent to about 0.8 days of typical OH oxidation, particle size distributions similar to fresh emissions were measured whereas at longer ageing times of a few days, more than an order of magnitude larger particle masses are observed. After about 5–6 eq. days of ageing, no significant further increases in the size distribution were observed for both fuels, indicating that the full particle mass formation potential was reached (Fig. 2A and B).

**Fig. 3:**
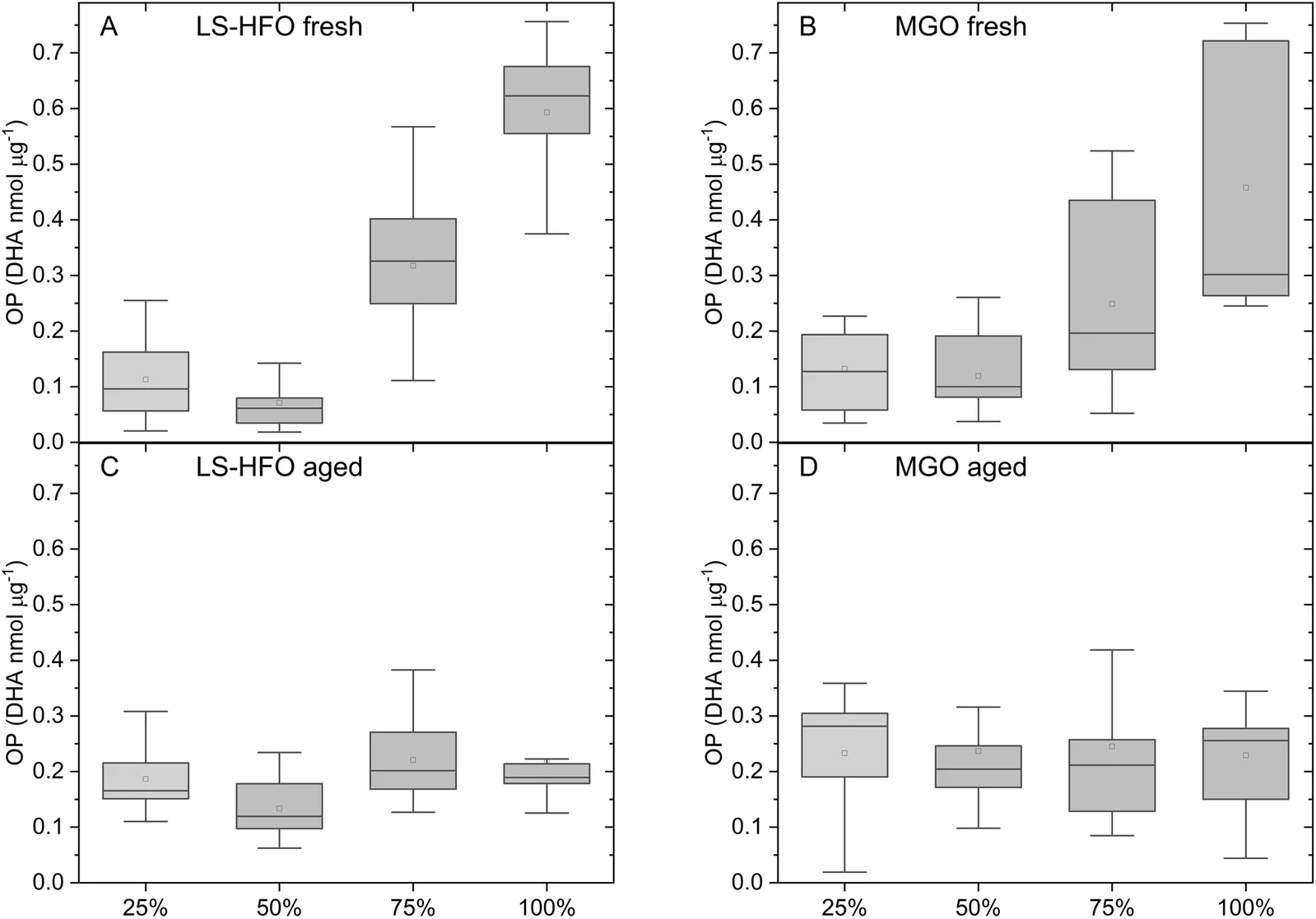
Changes of OP activity per µg PM mass of ship particle emissions at four different engine loads (see also Fig. S1) and for two different fuels. OP activity of fresh emissions (A) (LS-HFO), (B) (MGO), OP activity of aged emissions (C) (LS-HFO), (D) (MGO).

Fig. 3 shows mass normalized OP activity of PM emitted at different engine loads and for LS-HFO and MGO fuels as well as for fresh and aged conditions. Overall, OP values of both fuels varied over about an order of magnitude, from about 0.05 to 0.8 nmol DHA µg^−1^ (Fig. 3A–D). For the freshly emitted PM of both fuel types an increase in engine load results in an increased OP per PM mass by up to a factor of about ten (Fig. 3A and B). This trend as a function of engine load is not observed anymore in aged particles (Fig. 3C and D), where OP values are increased for low engine loads and decreased for high loads compared to fresh particles. The decrease of OP at higher loads might be due to more OP-inactive components in the secondary aerosol mass formed under high load experiments such as sulphate (see Table S3).

**Fig. 4:**
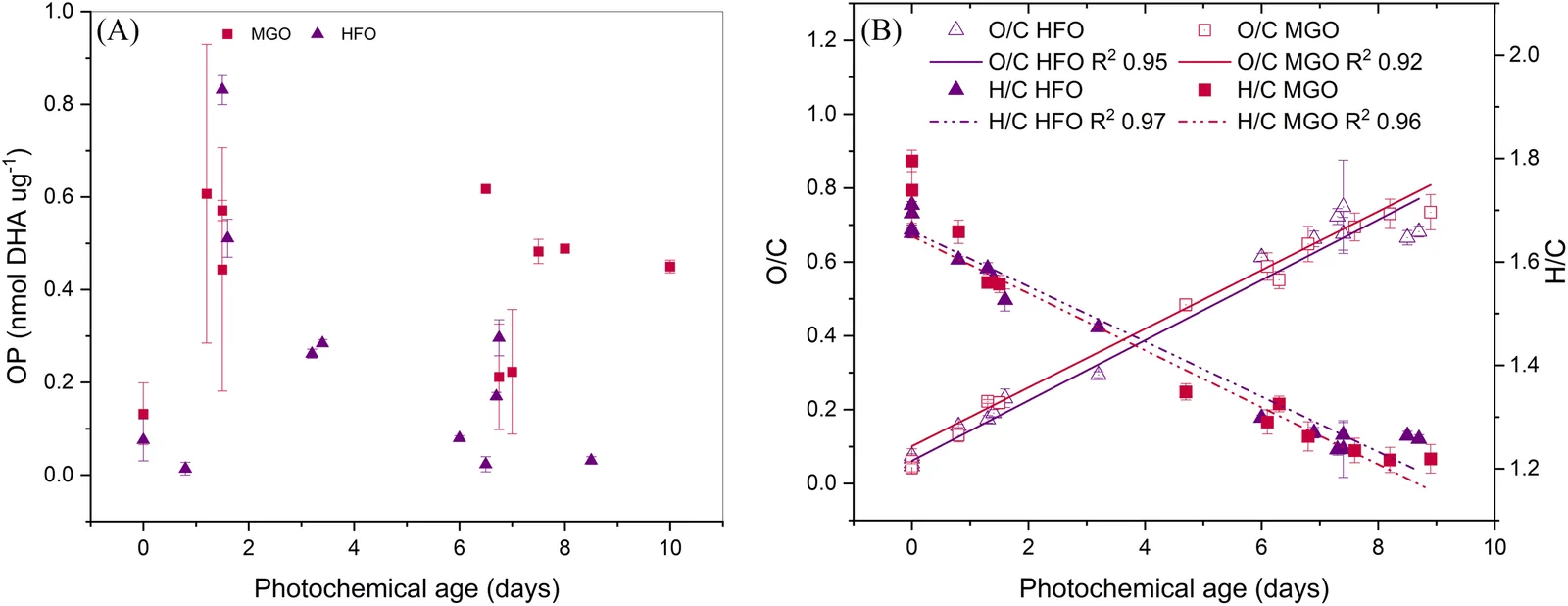
Change of OP (A) of ship emissions and two fuel types (MGO and LS-HFO) and of the average degree of oxidation of organic particle components (O/C and H/C, respectively) (B) with photochemical age for an equivalent of up to 10 eq. days atmospheric aging.

The ageing experiments presented in Fig. 3 were performed at constant O_3_ concentration and light intensities inside the PEAR. However, NO_*x*_ emissions varied for different engine loads resulting in different OH concentrations and thus different atmospheric ageing times. When the engine was running with MGO, the average photochemical age at 25% and 50% load was approximately 3.4 eq. days while at 75% and 100% load it was 1–1.2 eq. days (Fig. S4). When LS-HFO was used the ageing times were 0.5–1 day longer than for the respective MGO emissions (Fig. S4). A correction for these different ageing times was not attempted because OP formation rates are likely not linear across the aging times investigated here and because OP values did not change significantly between 1 and 3 days (see Fig. 4A).

Another set of experiments was performed where ageing time (*i.e.*, OH exposure) was varied between 1 and 10 eq. days atmospheric ageing at a constant engine load of 25% (thus keeping NO_*x*_ emissions constant). Under typical conditions, exposure to particles aged for about one day represent local-scale sources whereas exposure to particles aged for a week or more represent regional-scale sources.

Fig. 4A shows OP activity as a function of photochemical age for both fuels. No age-dependent trend in OP was observed for either fuel within experimental variability. OP activities for aged conditions were also not significantly higher than the fresh emissions (see data points at zero eq. days ageing time, Fig. 4A).

The average O/C of the organic mass fraction as measured by an AMS showed a linear increase throughout the range of ageing times illustrating the increased oxygen content in the aerosol and a decrease in the H/C ratio (Fig. 4B). These trends were identical for both fuels. Similarly, an increasing particle age resulted in a strong increase of sulphate content in the particles of both fuels (Table S3). In general, higher oxidized organic aerosol is known to be a contributor to aerosol toxicity,^54,55^ however, this is not reflected in the OP data, where no ageing-dependent trend was observed.

### Jet engine combustion chamber

**Fig. 5:**
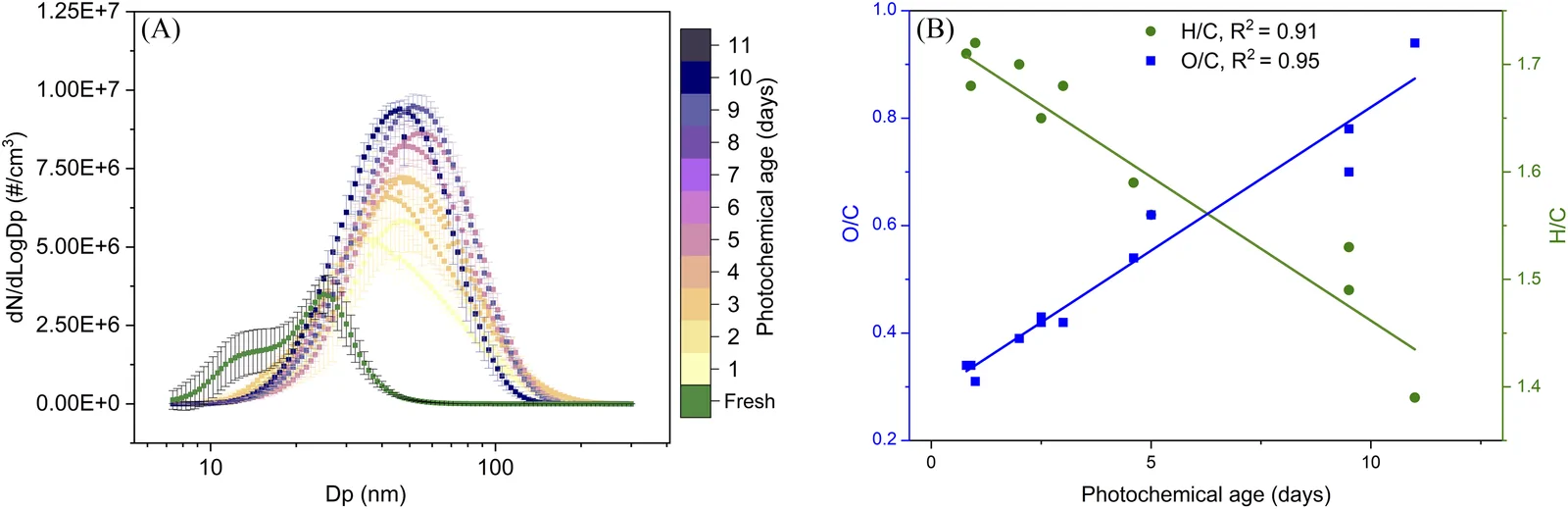
Particle number size distributions of fresh (green data) and aged aircraft emissions (A). Changes of the average degree of oxidation of organic particle components (H/C and O/C) with increasing photochemical age indicating the continuous oxidation for up to 11 eq. days of atmospheric aging (B).

The second type of aerosol particles investigated were from an aircraft combustion chamber running on JP-8 fuel to simulate aircraft emissions during the LTO cycle. Particle size distributions were measured of freshly emitted particles and aged up to 11 eq. days. The particle number and mass changed significantly between fresh emissions and aged conditions where increased number concentrations and a clear shift of the mode of the size distribution indicate new particle formation as well as a significant mass increase in the PEAR (Fig. 5A). A similar trend was observed with ageing of ship emissions (Fig. 2). However, for aircraft combustion chamber emissions, secondary particle mass growth was already observed at the lowest ageing times of about 1 day, in contrast to ship engine emissions, where short ageing times did not induce significant secondary particle formation.^56–58^

The overall chemical composition of the aged organic particle fraction, characterised with AMS, showed a continuously increasing O/C and a decreasing H/C, as expected of an aerosol exposed photochemical ageing (Fig. 5B). A very similar trend was observed for ship emissions (Fig. 4C), although higher O/C values were observed for aircraft combustion chamber particles after one day ageing, compared to ship emission particles. O/C values observed here compare well to SV-OOA and LV-OOA measured typically for SOA in chamber experiments and field campaigns.^59^

**Fig. 6:**
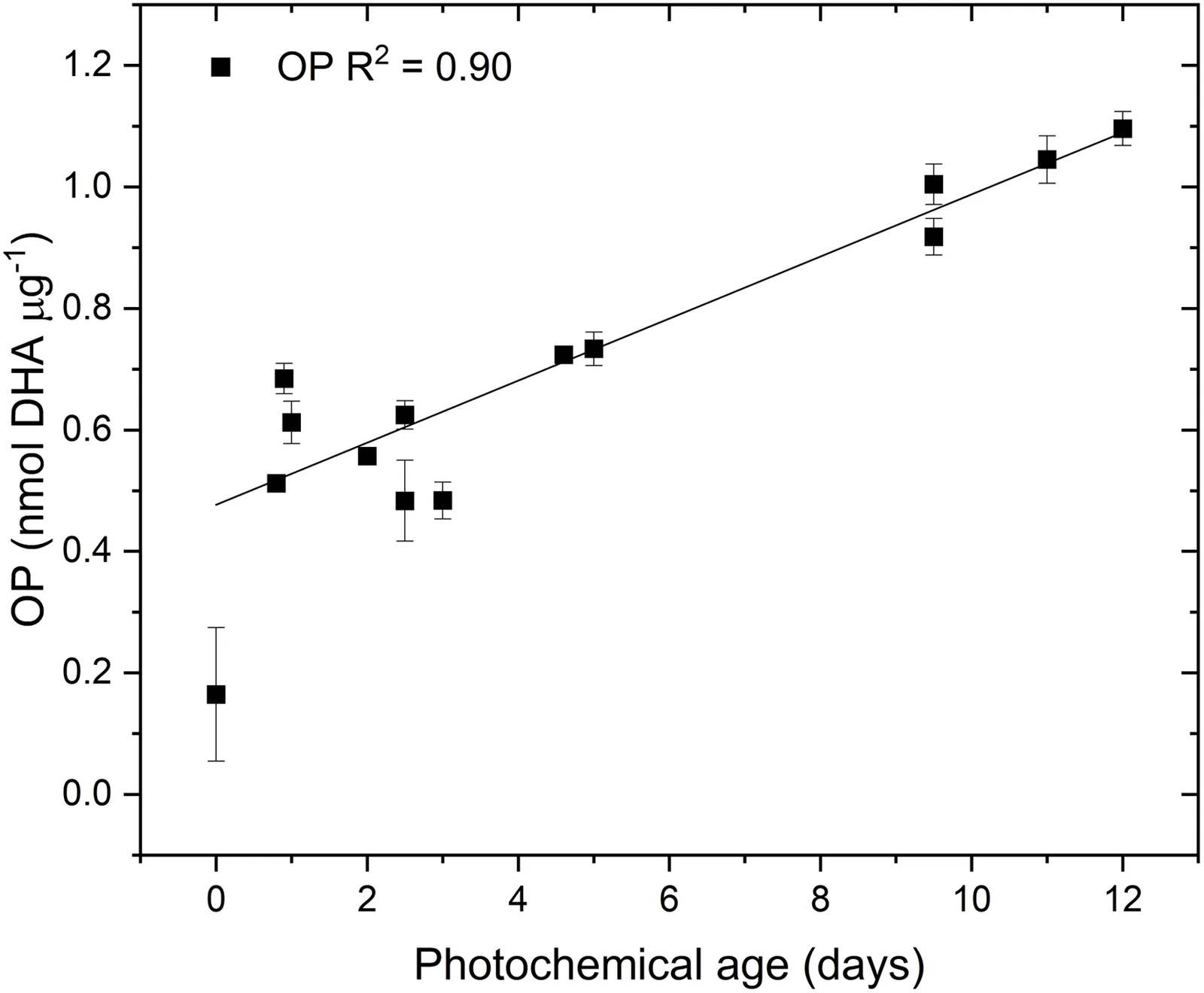
Changes in OP activity of aircraft particle emissions as a function of atmospheric aging up to 12 eq. days.

OP of the aircraft combustion chamber fresh and aged emission particles were compared for a fixed 7% load (Fig. 6). The fresh aircraft emission particles (*i.e.*, ageing time of zero eq. days in Fig. 6) had a similar OP activity than fresh ship emissions at a low load (Fig. 2). Increasing ageing time of aircraft emission particles caused OP to increase linearly (Fig. 6) which is in strong contrast to ship emissions. While no age-dependence was observed for ship emissions, a clear trend is visible for aircraft emissions (*R*^2^ of 0.9), increasing OP by a factor of six to seven between freshly emitted particles and aged for 12 eq. days (Fig. 6). The increase in sulphate in particles with increasing particle age (although less pronounced that for the ship emissions) might be related to this trend in OP (Table S3).

The contrasting behaviour of OP for aged ship and aircraft emissions (Fig. 4A and 6) potentially reflects the different OP-active components the assay is sensitive to. The OP assay is sensitive to redox-active species such as transition metals and quinone-like and other organic compounds. A more detail interpretation of this different aging trends of ship and aircraft emissions will require highly detailed molecular-level characterization of aged particles, which was not done in this study and would be an important characterization for a follow-up study.

### Online particle-bound ROS analysis

In addition to online OP data, another online instrument was deployed measuring reactive oxygen species, with the Online Particle-bound Reactive Oxygen Species Instrument (OPROSI), quantifying the sum of H_2_O_2_ and organic peroxides, as described in more detail previously.^39^ The ROS concentrations of the fresh emissions could not be determined accurately, possibly due to interference of high soot concentrations or other components in the engine exhaust samples interacting with a component in the ROS assay (*e.g.* HRP) causing slightly negative signal intensities as described in more detail elsewhere^60^ and in the SI. Despite these uncertainties, ROS signals of photo-chemical aged particles significantly increased for ship and aircraft emissions demonstrating that aging causes an increase of particle mass-normalized ROS (see Fig. S5 and related text).

## Conclusion

By applying a novel online instrument, we quantified OP in PM of two highly dynamic anthropogenic sources, the exhaust of a jet engine combustion chamber and a marine diesel engine. This online sampling and analysis technique avoids OP degradation compared to traditional offline analysis methods as illustrated elsewhere^35^ and thus reduce the risk of underestimating OP levels caused by the decomposition of short-lived OP- components in samples. Photochemical ageing caused no increase of OP activity in ship emissions while for aircraft emissions OP increased linearly with ageing, which was correlated with an increasing average oxidation of organic PM components as measured by the AMS.

Previous studies have reported positive relationships between aerosol oxidation state and OP activity. For example, Liu *et al.*, 2022 (ref. 61) observed enhanced cellular ROS production with increasing oxidation level of organic aerosol, and Tuet *et al.*, 2017 (ref. 62) reported increasing oxidative reactivity with photochemical ageing in chamber studies.

The absence of an ageing trend in AA-based OP for ship emissions suggests that particle oxidation state alone does not universally determine OP, and that source-specific composition and assay sensitivity play an important role. The specific reasons for the differences in increase of OP with aging of these engine types and fuels will require more detailed molecular-level analyses of emission particles in future studies. Unfortunately, for the set of experiments described here, such additional chemical particle composition data is not available.

Our findings suggest that PM emitted from sources in harbors and airports may differ in oxidative properties depending on source and atmospheric processing. Since oxidative reactivity is considered, a possible mechanistic pathway explaining PM-related health effects, relying solely on particle mass may not fully capture PM-related health effects. However, direct toxicological studies would be required to confirm implications for specific health outcomes. Our findings also highlight that oxidative PM properties of ship and aircraft emissions not only have potential impacts in the immediate vicinity of airports, harbours and busy shipping routes that are often close to population centres but also further away from these sources where emission plumes can still be detected.^63^

Current efforts to reduce emissions from the shipping sector focussing on SO_2_, NO_*x*_, and overall particle mass as well as greenhouse gases will lead to a significant emission reduction in the coming years. Besides technical adaptations like exhaust gas cleaning systems and scrubbers the use of low-carbon fuels are being considered.^62^ The results in this study illustrate that toxicity-specific particle properties such as OP need to be determined for new fuels or exhaust treatments and that especially atmospheric transformations of exhaust (*i.e.*, organic and inorganic components in the gas and particle phase) need to be taken into account to obtain a comprehensive assessment of transport emissions effects on human health. This study also highlights that more online OP are required to assess the magnitude and temporal variability of this health-relevant metric.^64^

Using the same online instruments to quantify OP and ROS, Utinger *et al.* 2025 (ref. 60) presented emission measurements from a gasoline car and a residential wood stove, whereas in this study ship engine and aircraft turbine emissions were characterised. OP activities of all fossil fuel combustion sources (car, ship, aircraft) were in a similar range when aged for about 3 eq. days (about 0.3 to 0.5 nmol DHA/mg for OP_M_ on average), whereas aged wood stove emissions were up to a factor of 10 times higher for OP_M_ (up to 3 nmol DHA/mg for OP_M_). This illustrates that PM originating from different combustions sources can have vastly different OP activities, especially when ageing processes in the atmosphere are considered. These data also highlight that combustion in a modern wood stove with controlled air supply (when operated without electrostatic precipitator) generates particles with about 1–2 orders of magnitude higher OP activities on a per particle mass basis than car, ship and aircraft emissions. This large difference in OP activities per PM mass between wood stove and engine emissions might be due the very different fuel types used, the differences in combustion conditions (*e.g.* batch mode addition of wood to the stove *versus* continuous and controlled addition of fuel for the engines) and different emission treatment technologies applied at the different sources. The ROS data (Fig. S5 and in Utinger *et al.* 2025 (ref. 60)) showed a similar trend with up to about 100 times higher ROS values for aged wood stove emissions compared to aged car, ship and aircraft emissions.

So far there are no other online OP or ROS emission data of combustion sources available in the literature. Campbell *et al.* 2025 (ref. 35) showed that a major fraction of OP and ROS decays within minutes to hours to a varying degree of 60 to 99% and depending on the particle type and composition. Thus, a comparison of the online data presented here with offline data is challenging. More online OP and ROS measurements are urgently needed to assess accurate levels of OP and ROS in different types of aerosol particles and to assess their importance for OP and ROS in ambient particles.

## Author contributions

A. B.: writing – original draft, visualization, formal analysis, investigation; B. U.: writing – review & editing, validation; Q. H., A. P., A. H.: investigation; M. I., S. P.: investigation, methodology; U. E., M. R., B. G.: conceptualization, investigation, methodology; T. S.: investigation, supervision, project administration; M. S.: investigation, supervision, methodology; H. C.: investigation, supervision; T. H., O. S.: conceptualization, funding acquisition, resources, supervision, project administration; Y. R., R. Z., J. Ø., T. A.: conceptualization, funding acquisition, resources, supervision; M. K.: conceptualization, supervision, writing – review & editing.

## Conflicts of interest

There are no conflicts to declare.

## Supplementary Material

EA-006-D5EA00174A-s001

EA-006-D5EA00174A-s002

EA-006-D5EA00174A-s003

## Data Availability

The data supporting this article have been included as part of the supplementary information (SI). Supplementary information: measured data provided as an excel file and additional data. See DOI: https://doi.org/10.1039/d5ea00174a.
