# Correction: Online characterisation of oxidative potential and reactive oxygen species of fresh and aged aircraft turbine and ship engine particulate emissions

**DOI:** 10.1039/d6ea90037b

**Published:** 2026-10-09

**Authors:** Alexandre Barth, Battist Utinger, Quanfu He, Andreas Paul, Anni Hartikainen, Mika Ihalainen, Uwe Etzien, Sandra Piel, Marius Rohkamp, Ajit Mudan, Benedikt Gündling, Tuukka Kokkola, Thorsten Streibel, Martin Sklorz, Hendryk Czech, Johan Øvrevik, Thomas Adam, Andreas Hupfer, Thorsten Hohaus, Olli Sippula, Yinon Rudich, Bert Buchholz, Ralf Zimmermann, Markus Kalberer

**Affiliations:** a Department of Environmental Science, University of Basel Basel Switzerland markus.kalberer@unibas.ch; b Institute for Climate and Energy Systems, ICE-3 Troposphere, Forschungszentrum Juelich GmbH Juelich Germany; c Department of Environmental and Biological Science, University of Eastern Finland Kuopio Finland; d Department of Piston Machinery and Internal Combustion Engines, University of Rostock Rostock Germany; e Institute for Chemistry & Environmental Engineering, University of the Bundeswehr Munich Neubiberg Germany; f Department of Technical and Analytical Chemistry, University of Rostock Rostock Germany; g Cooperation Group “Comprehensive Molecular Analytics”, Helmholtz Centre Munich Munich Germany; h Norwegian Institute of Public Health Oslo Norway; i Department of Chemistry, University of Eastern Finland Joensuu Finland; j Department of Earth and Planetary Science, Weizmann Institute of Science Rehovot Israel; k Institute for Aeronautical Engineering, University of the Bundeswehr Munich Ottobrunn Germany

## Abstract

Correction for “Online characterisation of oxidative potential and reactive oxygen species of fresh and aged aircraft turbine and ship engine particulate emissions” by Alexandre Barth *et al.*, *Environ. Sci.: Atmos.*, 2026, https://doi.org/10.1039/d5ea00174a.

The authors regret that the name of one of the authors, Tuukka Kokkola, was presented incorrectly in the original article. The corrected list of authors for this paper is as shown here.

In addition, the authors regret that one of the affiliated institutions (Department of Environmental and Biological Science, University of Eastern Finland, Kuopio, Finland) was duplicated (^*c*,*i*^) in the original article. The correct affiliations order for this paper are as shown here.

The Royal Society of Chemistry apologises for these errors and any consequent inconvenience to authors and readers.

