# Supplementary material for: Online characterisation of oxidative potential and reactive oxygen species of fresh and aged aircraft turbine and ship engine particulate emissions
Source: Environ Sci Atmos. 2026 Sep 22;6(10):1629–38. doi: 10.1039/d5ea00174a (PMC13596030; doi:10.1039/d5ea00174a)
Supplement: Supplementary file 1 [file EA-006-D5EA00174A-s002.pdf]

Supplementary Information (SI) for Environmental Science: Atmospheres.
This journal is © The Royal Society of Chemistry 2026

Supplement Information

Online Characterisation of Oxidative Potential and Reactive Oxygen Species of Fresh and Aged Aircraft Turbine and Ship Engine Particulate Emissions

Alexandre Barth, Battist Utinger, Quanfu He, Andreas Paul, Anni Hartikainen, Mika Ihalainen, Uwe Etzien, Sandra Piel, Marius Rohkamp, Ajit Mudan, Benedikt Gündling, Kokkola Tuuka, Thorsten Streibel, Martin Sklorz, Hendryk Czech, Johan Øvrevik, Thomas Adam, Andreas Hupfer, Thorsten Hohaus, Olli Sippula, Yinon Rudich, Bert Buchholz, Ralf Zimmermann, Markus Kalberer

Table S1: Fuel properties of the two ship engine fuels using in this study.

Fuel property	MGO	LS-HFO
Viscosity at 50 °C (mm^2/s)	2.794	123.420
Density (g/cm^3)	0.8516 at 15 °C	0.9065 at 50 °C
Water content (ppm)	93	659
Sulfur content (mg/kg)	150	5060

Table S2: Overview of the exhaust dilutions applied during the different measurement (see Figure 1).

Source	Measurement	Dilution ratio
Ship	experiments where engine load was changed (data in Figure 3)	1:100
Ship	aging experiment (data in Figure 4)	1:250
Aircraft	aging experiment (data in Figure 6)	1:200

Table S3: AMS measurements of organics to sulphate ratio.

Ship HFO		Ship MGO		Jet fuel	
Aging (days)	Org / SO_4 ratio	Aging (days)	Org / SO_4 ratio	Aging (days)	Org / SO_4 ratio
0.0	68.0	0.0	77.5	0.0	316.0
1.4	5.0	1.5	13.9	0.9	81.0
3.0	1.5	4.0	2.9	2.5	52.0
6.0	0.5	6.0	2.2	4.6	32.0
7.0	0.4	7.0	2.3	11.0	18.0
8.0	0.4	7.5	1.7		
9.0	0.4	8.0	1.5		
		10.0	1.6		

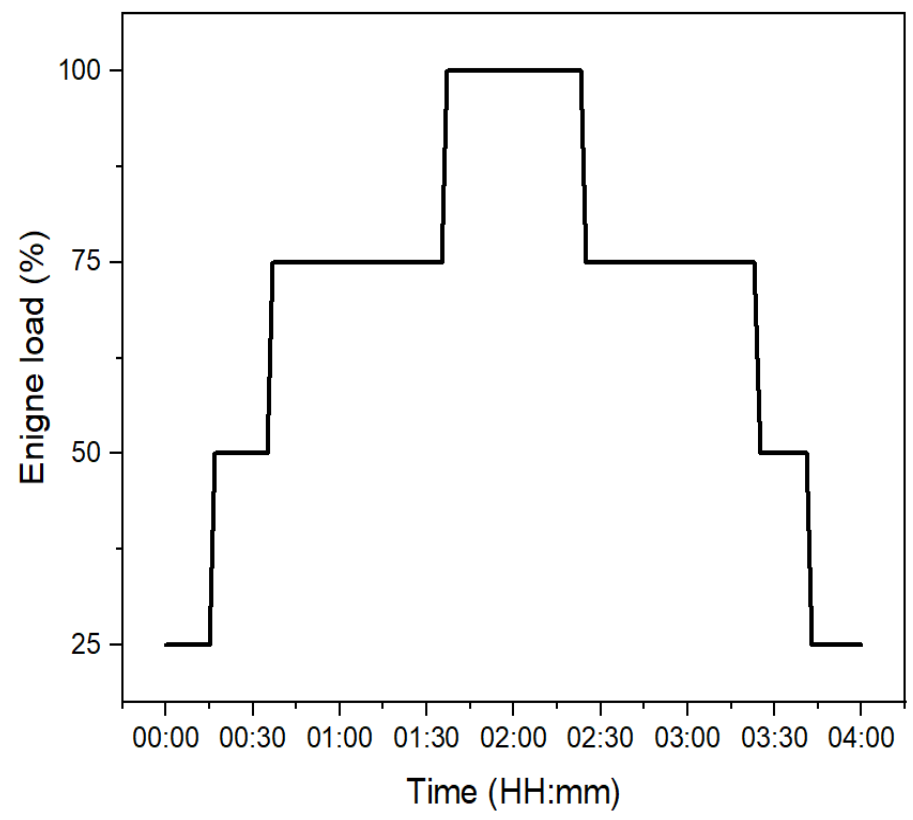

Figure S1: Load cycle of the ship engine during a 4-hour experiment.

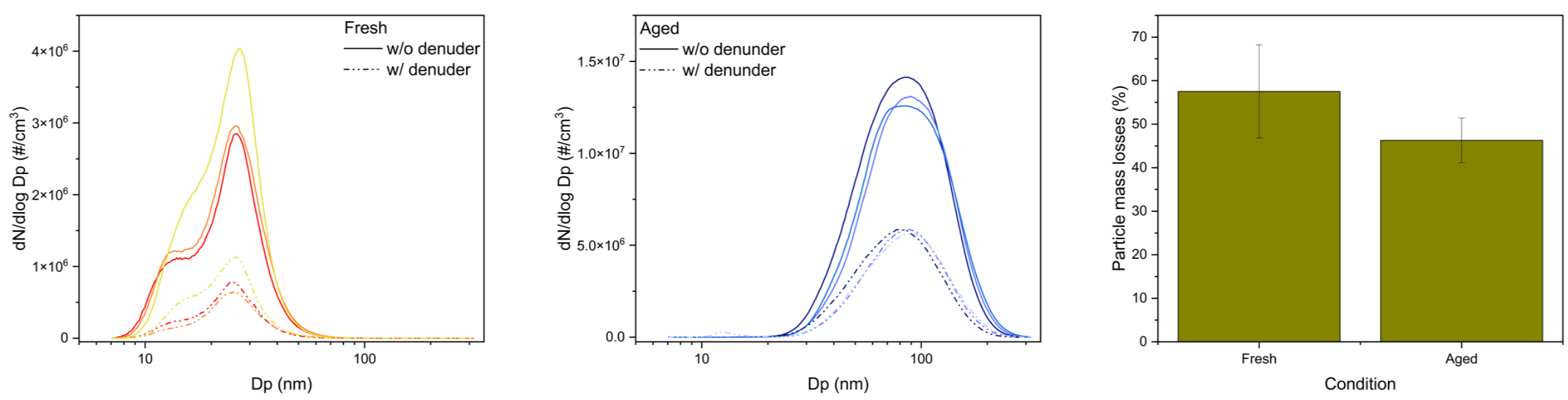

Figure S2: Determination of particle losses in the charcoal denuders. These losses were used to correct the mass normaised OP and ROA activity data. Particle size distributions were determiend with an SMPS measuring before and after the charcoal denuders during aircraft measurements.

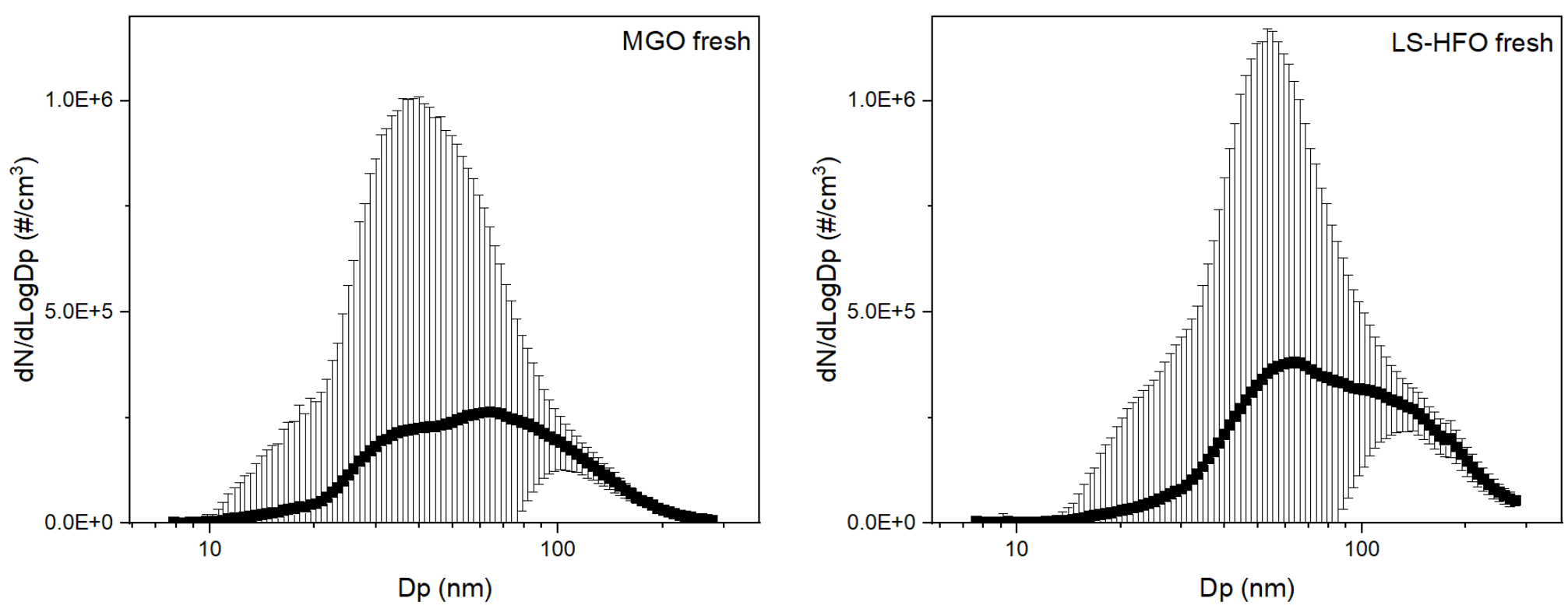

Figure S3: Particle size distributions for fresh MGO and LS-HFO ship emissions at 25% enging load.

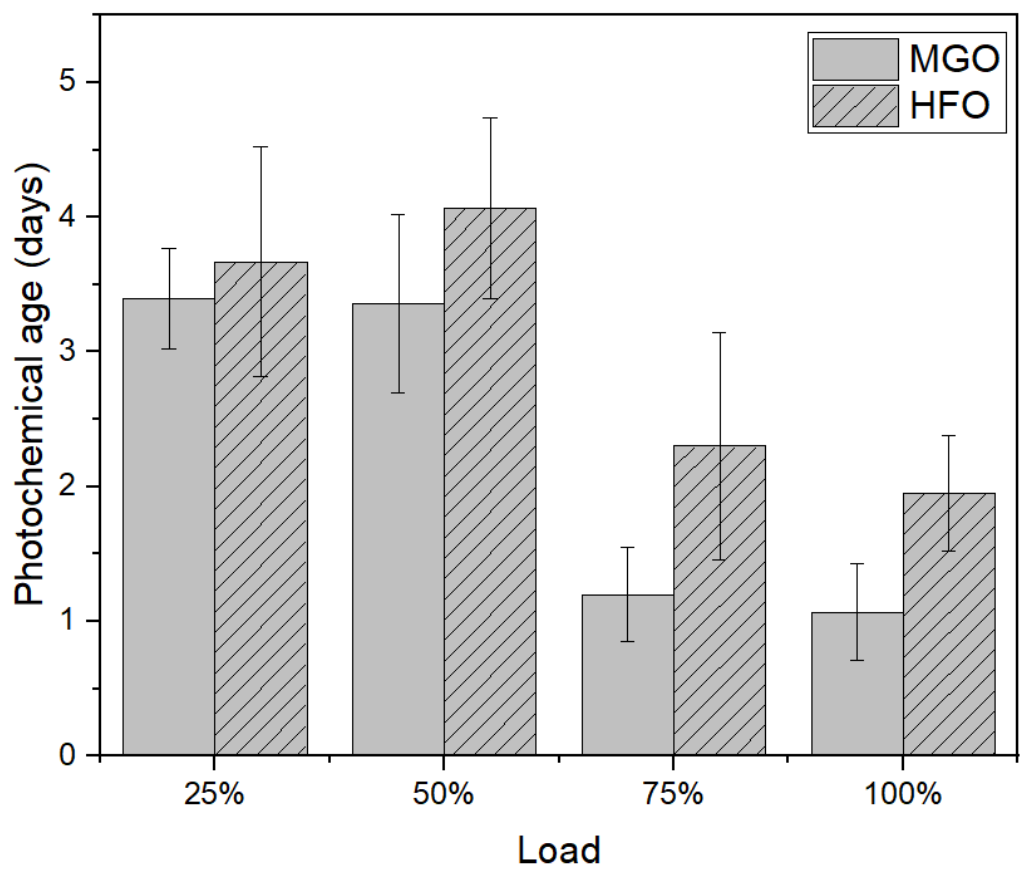

Figure S4: Average photochemical ages during the load cycle of the ship engine for MGO and LS-HFO.

ROS Measurements

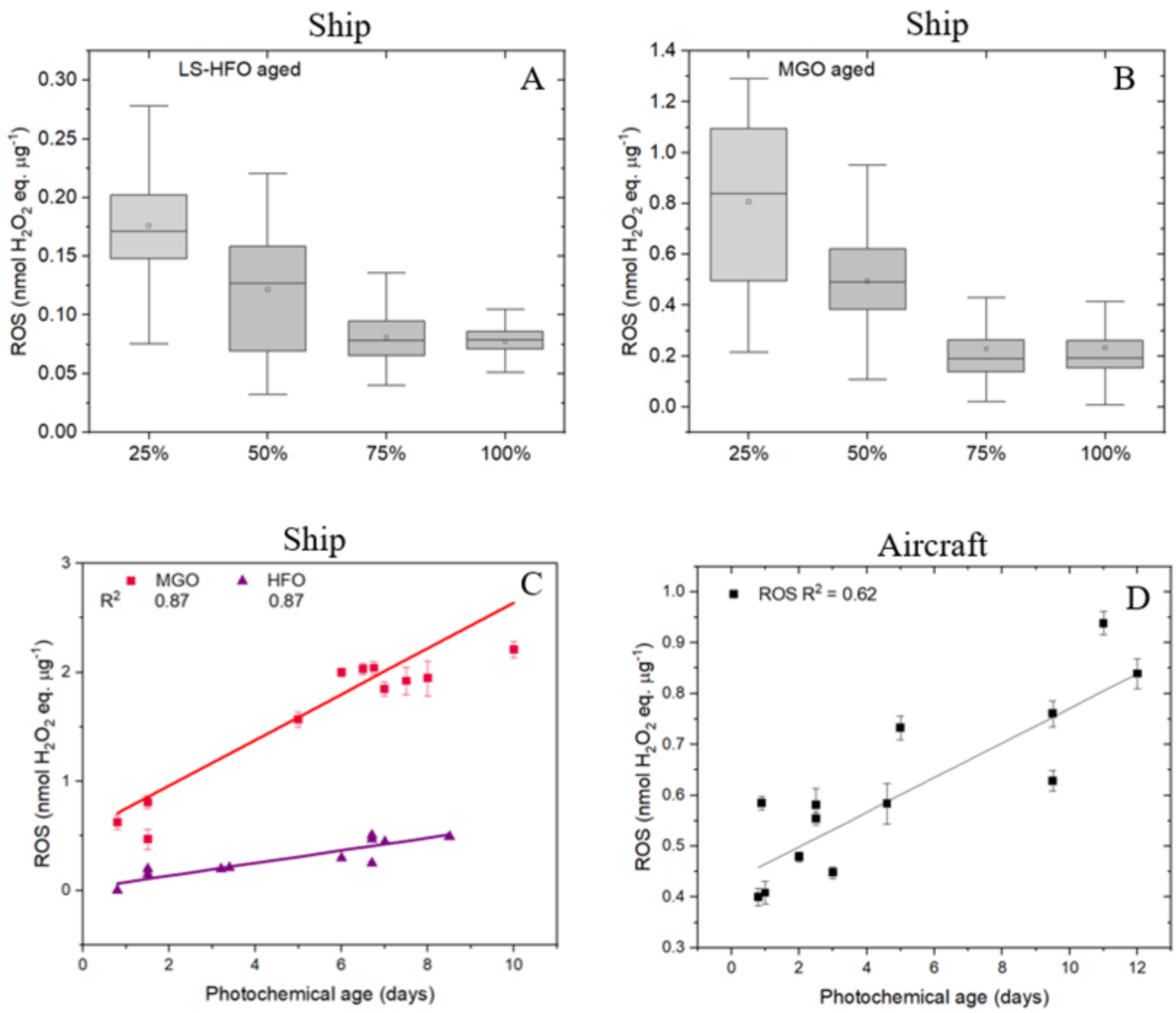

Figure S5: ROS concentrations in ship engine emission particles when operated with LS-HFO, aged (A), and with MGO, aged (B). Change in ROS concentration of ship emissions and two fuel types (MGO and LS-HFO) (C) and in ROS concentration of aircraft particle emissions as a function of atmospheric aging from one up to 12 eq. days (D).

As described in the method section (see main text) in addition to online OP measurements, online ROS measurements were performed.

Figures S5A and S5B show ROS concentrations of ship engine emissions for both fuels (MGO and LS-HFO) and for different engine loads. ROS values increase slightly at lower engine loads. MGO data were about a factor of 3-5 larger than LS-HFO data.

Figure S5C illustrates that ROS concentrations increased linearly by a factor of about three for MGO when particles were photochemical aged between 1 and 10 eq. days. A similar increase was measured for LS-HFO, although at much lower absolute values. This continued increase in ROS till 10 eq. days of ageing contrasts with the evolution of particle size distribution and SOA mass formation, which levels off after about 6 eq. days of ageing, indicating that a compositional change is still taking place after SOA mass increase ceased.

In figure S5D ROS concentrations of the aircraft combustion chamber are shown for aged emission particles at a fixed engine load of 7%. Increasing ageing time caused ROS to increase

linearly. ROS concentrations increased by a factor of about two when ageing time increased from 0.8 to 12 eq. days (R^2 of 0.62), which is in a similar range as the ship emissions, although a strong differences between MGO an LS-HFO was observed (figure S5C).

For both engines and fuel types, ROS values for fresh emissions (i.e., no oxidative aging) could not be determined reliably as measurements resulted in values slightly below blank values. This is likely an artifact of the chemical assay in the online ROS instrument where horseradish peroxidase (HRP) might become deactivated in the presence of very high soot particle concentrations in the sample, as previously described[1]. A quantification and correction of this artifact is for the current data set not possible and thus also ROS data of aged emissions presented in figure S5 should be considered as preliminary.

References

[1] B. Utinger *et al.*, “Emission dynamics of reactive oxygen species and oxidative potential in particles from a petrol car and wood stove,” *Aerosol Res.*, vol. 3, no. 1, pp. 205–218, Apr. 2025, doi: 10.5194/ar-3-205-2025.
